# “Falls in older people receiving home healthcare services at armed forces hospital Southern Region: prevalence, associated factors, and consequent injuries”

**DOI:** 10.1186/s12877-026-07302-3

**Published:** 2026-03-13

**Authors:** Ali M. Al Qahtani, Sadia Aslam, Osama M. A. Ali, Shahinaz Y. Abd EL-Rahim, Hiba A. Mamoun, Rasha A. M. Ali

**Affiliations:** 1 Department of Home Health Care, Armed Forces Hospital Southern Region, Khamis Mushait, Saudi Arabia; 2 Department of Geriatrics and Gerontology, Faculty of Medicine, Ain Shams University, Cairo, Egypt

**Keywords:** Falls in older adults, Home health care, fall risk assessment, Falls related Injuries

## Abstract

**Background:**

Falls are a major cause of morbidity and loss of independence among older adults, particularly those receiving home healthcare services due to advanced age, multimorbidity, and environmental hazards. Despite the expansion of Home Health Care (HHC) services in Saudi Arabia, evidence regarding fall prevalence, associated factors, and home-related hazards among HHC recipients remains limited.

**Objectives:**

To determine the prevalence of falls and related injuries and to examine medical, medication-related, and environmental factors associated with falls among older adults receiving HHC services at the Armed Forces Hospital, Southern Region.

**Methods:**

A cross-sectional study was conducted over three months among adults aged ≥ 65 years enrolled in the HHC program. A total of 368 participants were selected using systematic random sampling. Data were collected through structured interviews, medical record review, the Missouri Alliance for Home Care 10-Fall Risk Assessment Tool, and a home safety evaluation using the CDC STEADI tool. Descriptive and inferential statistics (Chi-square test, Fisher’s exact test, and independent t-test) were applied, with statistical significance set at *p* < 0.05.

**Results:**

The 12-month prevalence of falls was 20.9%; most participants who reported falls experienced a single event (76.6%), and fractures were reported in 2.4%. Stroke (*p* = 0.009) and depression (*p* = 0.024) were significantly associated with fall occurrence. Antipsychotics (*p* = 0.009) and antidepressants (*p* < 0.001) were significantly associated with falls. Higher fall-risk assessment scores were also significantly associated with fall occurrence (*p* = 0.001). Environmental hazards, particularly broken or uneven steps and absence of handrails, demonstrated significant associations with falls.

**Conclusion and recommendations:**

Falls among older adults receiving HHC services were significantly associated with specific comorbidities, psychotropic medication use, higher fall-risk scores, and hazardous home environmental conditions. These findings highlight the importance of targeted fall-prevention interventions focusing on potentially modifiable associated factors to enhance safety in this population.

**Supplementary Information:**

The online version contains supplementary material available at 10.1186/s12877-026-07302-3.

## Introduction

Falls are a leading cause of injury and loss of independence in older adults worldwide. Globally, approximately three in ten adults aged ≥ 65 years experience at least one fall each year, with risk rising further after age 70. Ongoing demographic ageing will amplify this burden: the share of the global population aged ≥ 65 years is projected to increase from ~ 10% in 2022 to ~ 16% by 2050 (“one in six people”), ensuring that fall-related morbidity, mortality, and costs continue to climb [1–4].

Falls result in fractures, head trauma, functional decline, depression, fear of falling, reduced mobility, and institutionalization, imposing substantial direct and indirect costs on health systems. In the United States, more than one in four older adults falls annually, and a prior fall doubles the risk of a subsequent fall. Although international guidance emphasizes that many falls are preventable, their physical and psychological consequences often persist, and many older people do not recover to their pre-fall functional level [5–7].

In Saudi Arabia, recent studies report a high prevalence of falls among community-dwelling older adults, with city-level estimates ranging from ~ 21% in Riyadh to ~ 47% in Jeddah; injuries and fractures are common among those who fall. A 2025 nationwide cross-sectional study identified important associated factors and highlighted the scarcity of comprehensive national data to inform prevention strategies [8–10]. However, these studies were conducted primarily among community-dwelling older adults and did not specifically examine individuals receiving structured home healthcare services, who may differ substantially in clinical complexity, functional dependency, and environmental exposure.

Despite the expansion of home healthcare (HHC) services for older Saudis, peer-reviewed evidence on falls among HHC recipients remains limited. In Saudi Arabia, home healthcare services are primarily hospital-based and physician-led, often attached to tertiary or military healthcare institutions, and are delivered within a healthcare system undergoing rapid demographic transition and service restructuring under Vision 2030. Unlike some high-income countries where community-based geriatric services are long-established and multidisciplinary home modifications are systematically integrated, Saudi HHC services are still evolving, with variability in standardized fall-risk assessment, environmental modification practices, and caregiver training across regions.

Older adults receiving HHC represent a clinically distinct and more vulnerable subgroup characterized by higher levels of functional dependency in activities of daily living, increased frailty, multiple chronic conditions, polypharmacy, cognitive impairment in some cases, and prolonged exposure to home environmental hazards. In the Saudi context, additional factors may further influence fall patterns, including multigenerational living arrangements, reliance on informal family caregivers, variability in housing design (e.g., multi-level homes with marble or tiled flooring), and climate-related environmental features that may affect indoor safety. Unlike relatively independent community-dwelling older adults, HHC recipients often spend the majority of their time within the home environment, which may amplify the impact of structural hazards such as uneven flooring, poor lighting, and absence of assistive modifications. Unlike community-based studies, older adults enrolled in HHC programs typically have higher levels of multimorbidity, polypharmacy, functional impairment, and home-based environmental risks, potentially placing them at a distinct fall profile.

In the Southern Region, an observational study described fall-prevention knowledge and practice patterns among HHC professionals, underscoring the need to translate awareness into standardized assessment and targeted, home-based interventions [11]. However, that study focused on healthcare providers rather than patient-level fall prevalence, injury patterns, or associated medical and environmental factors. Moreover, national data on fall epidemiology in Saudi Arabia have largely focused on community-dwelling older adults, with limited examination of institutional or structured home healthcare populations, leaving uncertainty regarding whether international fall-prevention models are directly transferable to the Saudi HHC system.

Accordingly, a critical evidence gap remains: to date, no study in the Southern Region—and very limited research nationally—has comprehensively quantified fall prevalence, related injuries, and the spectrum of medical, medication-related, and home environmental factors associated with falls specifically among older adults actively receiving HHC services. Conceptualizing HHC recipients as a distinct high-risk clinical population underscores the importance of generating setting-specific evidence rather than extrapolating findings from community-dwelling cohorts. Given Saudi Arabia’s rapidly aging population, ongoing health system transformation, and expanding hospital-affiliated HHC programs, generating context-specific evidence is particularly important to inform service planning, resource allocation, and culturally appropriate fall-prevention strategies. Addressing this gap is essential, as extrapolating findings from community-dwelling populations may not accurately reflect the risk profile of HHC recipients.

Given the cross-sectional nature of the present study, the objective is not to establish causal risk relationships but to examine statistical associations between falls and selected medical conditions, medication use, fall-risk assessment scores, and home environmental characteristics.

This study aims to determine the prevalence of falls and consequent injuries and to assess factors associated with fall occurrence, particularly potentially modifiable comorbidities, medications, and environmental hazards, among older adults receiving home healthcare services at the Armed Forces Hospital, Southern Region (Saudi Arabia).

## Subjects & methods

### Study design and setting

This study employed a cross-sectional design and was conducted over a period of three months at the Home Health Care Department, Armed Forces Hospital, Southern Region (AFHSR), Saudi Arabia.

AFHSR is a tertiary-care military hospital that primarily provides healthcare services to military personnel, retirees, and their eligible family members in the Southern Region of Saudi Arabia. The hospital offers specialized and referral services and operates structured home healthcare (HHC) programs for clinically complex and functionally dependent older adults.

The Home Health Care Department delivers physician-led, multidisciplinary services, including nursing care, medication management, chronic disease monitoring, and home-based functional assessment. Patients enrolled in this program typically represent a medically complex population characterized by advanced age, multimorbidity, functional dependency, and frequent healthcare utilization.

Given the cross-sectional nature of the study, the analysis was designed to assess statistical associations between fall occurrence and selected medical, medication-related, and environmental factors, rather than to establish temporal relationships or causal inferences.

### Study population

The study population included older adults aged 65 years and above actively enrolled in the hospital’s home healthcare program.

At the time of the study, the HHC program served a defined cohort of older beneficiaries residing within the hospital’s catchment area. The majority of patients were retired military personnel or their dependents, and many had long-standing chronic conditions requiring ongoing medical supervision.

Inclusion Criteria: Older adults aged ≥ 65 years receiving Home Health Care services at AFHSR and participants or legal guardians willing to provide written informed consent.

Exclusion Criteria: Bedridden elderly patients unable to undergo the assessment and refusal to participate.

Because the study was conducted within a military-affiliated tertiary hospital system, the sociodemographic and clinical profile of participants may differ from that of older adults receiving care in Ministry of Health or private-sector facilities. This context should be considered when interpreting the generalizability of the findings.

### Sample size and sampling technique

The sample size was calculated using OpenEpi Version 3, based on a falls prevalence of 48% reported by *Almarwani et al.*,* 2024* [12]. With a 95% confidence interval, a 5% margin of error, and a design effect of 1, the required sample size was 384 elderly participants.

The design effect of 1 was applied because the sampling frame consisted of an individual-level registry of patients enrolled in the Home Health Care program, and participants were selected independently using systematic random sampling. Each participant represented a single household, and no clustering by household or caregiver was present in the sampling structure. Therefore, the assumption of independence between observations was considered appropriate, and adjustment for clustering was not required.

During data collection, 384 eligible participants were approached; however, 16 were excluded due to incomplete data and non-response, resulting in a final analyzed sample of 368 participants (response rate: 95.8%). A post-hoc consideration indicates that the achieved sample size of 368 remains close to the initially calculated sample and is expected to retain adequate statistical power to detect moderate associations at the 5% significance level, with only a minimal and unlikely impact on study precision.

A systematic random sampling technique of eligible home healthcare patients was applied.

### Data collection tools and procedures

Data were collected via face-to-face interviews, home-based assessments, and review of medical records. The following tools were used:

1. Fall Screening Form: Collected demographic data, medical history, comorbidities, medication use, and fall events. Falls were defined using the WHO definition as “an inadvertent coming to rest on the ground, floor, or lower level, excluding intentional position changes” [1].

Fall history over the preceding 12 months was obtained primarily through structured interviews with participants. In cases where participants had documented cognitive impairment (e.g., dementia) or were unable to provide reliable responses, information was obtained from primary caregivers and cross-checked, when available, with medical records documented in the Home Health Care files to enhance data accuracy.

2. Medication Review Checklist: Documented all current medications, with particular focus on high-risk categories such as antidepressants, antipsychotics, antihypertensives, and diuretics.
3. Missouri Alliance for Home Care’s 10-Fall-Risk Assessment Tool: A structured fall-risk scoring tool was used to quantify fall-risk level. This tool has been applied in home healthcare settings as a structured instrument for identifying multifactorial fall risk among older adults and aligns with evidence-based recommendations for standardized fall-risk screening in geriatric populations [13]. The tool classified participants into Low, Medium, and High-risk categories. The original scoring system and cut-off thresholds recommended by the developers were applied without modification. The score incorporated multiple fall-related risk factors and generated a numerical risk score used in the analysis.
4. Home Safety Assessment (STEADI Tool Kit – CDC): Evaluated environmental hazards in key household areas: stairs, floors, bathrooms, bedrooms, and the kitchen. The STEADI (Stopping Elderly Accidents, Deaths, and Injuries) initiative, developed by the U.S. Centers for Disease Control and Prevention (CDC), is supported by international guidelines as an evidence-based framework for fall-risk screening and environmental safety assessment [14–15]. The standard STEADI home safety checklist and its recommended criteria were used without alteration.

### Validity and reliability

The Missouri Alliance for Home Care’s 10-Fall Risk Assessment Tool and the CDC STEADI Home Safety Tool Kit are validated tools widely used in geriatric fall-risk evaluation [13–14].

A pilot test was conducted on 40 patients to ensure feasibility and clarity; these data were excluded from the final analysis. The pilot phase confirmed the clarity of scoring procedures and the applicability of the original cut-off classifications in the local context without requiring modification.

### Statistical analysis

Data analysis was performed using IBM SPSS software (version 20). Descriptive statistics summarized participant characteristics and fall prevalence. Inferential statistics included: Chi-square or Fisher’s exact test for categorical data & Independent-samples t-test for continuous variables. A p-value < 0.05 was considered statistically significant.

## Results

The study participants were predominantly old–old (75–84 years; 41.8%) with a large oldest-old segment (≥ 85 years; 35.3%). The mean age was 82.1 ± 9.83 years (range 65–122), and 71.2% were female. Falls in the past 12 months were reported by 20.9% of participants. Among those who fell (*n* = 77), 76.6% experienced a single fall and 23.4% reported recurrent falls (≥ 2). At the population level, the post-fall injury burden was low: any injury, 5.2%; fracture, 2.7% (Table 1).

**Table 1:** Participant characteristics and fall history in the past year (*n* = 368)

Variable	Mean ± SD	Range
Age (years)	82.10 ± 9.83	65–122

**Variable**	**Category**	***n*** ** (%)**
Age Group	65–74 years	84 (22.8)
	**75–84 years**	154 (41.8)
	**≥ 85 years**	130 (35.3)
Sex	**Male**	106 (28.8)
	Female	262 (71.2)
Fall History (Past 12 Months)	No	291 (79.1)
		77 (20.9)
Falls Frequency (ǂǂ)	1 time	59 (76.6)
	2 times	8 (10.4)
	3 times	6 (7.8)
	**>** ** 3 times**	4 (5.2)

(ǂǂ) Falls frequency calculated among participants who reported falls (*n* = 77)

Concerning Prevalence of Comorbidities Among Participants; the current study reveals a heavy cardiometabolic burden—hypertension (80.4%) and diabetes mellitus (74.5%)—with substantial cardiovascular morbidity, including ischemic heart disease (28.5%), atrial fibrillation (14.4%), cardiomyopathy (7.1%), valvular disease (6.2%), and prior stroke (26.1%). Non-cardiac comorbidities are also frequent: chronic kidney disease (21.7%), osteoarthritis (22.8%), osteoporosis (13.0%), hypothyroidism (13.6%), depression (14.1%), dementia (21.2%), seizure disorders (5.2%), and interstitial lung disease/COPD (10.3%). Sensory and functional impairments are common—visual impairment (37.5%), hearing impairment (22.0%), and urinary incontinence (46.5%)—whereas fecal (stool) incontinence (7.1%) is less prevalent. Several conditions are relatively uncommon in this cohort—other arrhythmias (0.8%), hyperthyroidism (1.1%), dizziness/vertigo (1.1%), deep-vein thrombosis (1.9%), anemia (3.8%), Parkinson’s disease (4.9%), and old fractures (4.9%)—highlighting an overall profile dominated by high burdens of hypertension, diabetes, vascular disease, and age-related sensory/functional impairment (Table 2).

**Table 2:** Prevalence of comorbidities among participants (*n* = 368)

Comorbidity	*n* (%)
Diabetes Mellitus (DM)	274 (74.5)
Hypertension (HTN)	296 (80.4)
Chronic Kidney Disease (CKD)	80 (21.7)
Ischemic Heart Disease (IHD)	105 (28.5)
Atrial Fibrillation (AF)	53 (14.4)
Arrhythmia	3 (0.8)
Cardiomyopathy	26 (7.1)
Hypothyroidism	50 (13.6)
Hyperthyroidism	4 (1.1)
Anemia	14 (3.8)
Valvular Heart Disease	23 (6.2)
Stroke	96 (26.1)
Parkinson’s Disease	18 (4.9)
Dementia	78 (21.2)
Depression	52 (14.1)
Seizure Disorder	19 (5.2)
Dizziness	4 (1.1)
Deep Vein Thrombosis (DVT)	7 (1.9)
Osteoporosis	48 (13.0)
Osteoarthritis	84 (22.8)
Visual Impairment	138 (37.5)
Hearing Impairment	81 (22.0)
Urinary Incontinence	171 (46.5)
Stool Incontinence	26 (7.1)
Chronic Obstructive Pulmonary Disease (COPD)	38 (10.3)
Previous Fracture	18 (4.9)

Regarding Post-Fall Complications among Study Participants, the present study shows that most participants could not stand after the fall (90.2%). Serious complications were uncommon: fracture occurred in 2.4%, hematoma in 1.4%, hospitalization in 0.8%, and no subdural hematomas were recorded. Overall, the profile indicates a low observed rate of acute medical complications following falls in this sample, despite the high proportion unable to stand immediately post-fall—highlighting the need for prompt assessment and support after falls even when major injuries are infrequent (Table 3).

**Table 3:** Frequency of post-fall complications among study participants (*n* = 368)

Post-Fall Outcome	*n* (%)
Unable to Stand	36 (9.8)
Fracture	9 (2.4)
Hematoma	5 (1.4)
Subdural Hematoma	0 (0.0)
Hospitalization	3 (0.8)

The present study found that only a few comorbidities are significantly associated with falls: prior stroke (OR = 2.020, 95% CI 1.182–3.451; *p* = 0.009) and depression (OR = 2.072, 95% CI 1.088–3.946; *p* = 0.024) are linked to higher odds of falling. Most other conditions—including diabetes, hypertension, CKD, IHD, thyroid disorders, anemia, valvular disease, osteoporosis/osteoarthritis, sensory impairments, incontinence, COPD, seizures, dizziness, DVT, and old fractures—show no statistically significant association with falls (all *p* > 0.05) (Table 4).

**Table 4:** Association between comorbidities and falls (*n* = 368)

Comorbidity	No Falls *n* (%)	Falls *n* (%)	Odds Ratio (95% CI)	*P*-value (#)
Diabetes Mellitus	72 (76.6)	22 (23.4)	0.822 (0.469–1.441)	0.493
Hypertension	58 (80.6)	14 (19.4)	1.120 (0.587–2.138)	0.731
Chronic Kidney Disease	233 (80.9)	55 (19.1)	1.607 (0.907–2.847)	0.102
Ischemic Heart Disease	208 (79.1)	55 (20.9)	1.002 (0.575–1.748)	0.993
Atrial Fibrillation	243 (77.1)	72 (22.9)	0.352 (0.135–0.916)	0.026*
Arrhythmia	290 (79.5)	75 (20.5)	7.733 (0.692–86.438)	0.050*
Cardiomyopathy	269 (78.7)	73 (21.3)	0.670 (0.224–2.005)	0.471
Hypothyroidism	254 (79.9)	64 (20.1)	1.394 (0.700–2.777)	0.342
Hyperthyroidism	287 (78.8)	77 (21.2)	0.412 (0.022–7.739) (†)	0.301
Anemia	281 (79.4)	73 (20.6)	1.540 (0.469–5.050)	0.503
Valvular Heart Disease	269 (78.0)	76 (22.0)	0.161 (0.021–1.213)	0.059
Stroke	224 (82.4)	48 (17.6)	2.020 (1.182–3.451)	0.009**
Parkinson Disease	275 (78.6)	75 (21.4)	0.458 (0.103–2.038)	0.385
Dementia	228 (78.6)	62 (21.4)	0.876 (0.467–1.643)	0.679
Depression	256 (81.0)	60 (19.0)	2.072 (1.088–3.946)	0.024*
Seizure Disorder	276 (79.1)	73 (20.9)	1.008 (0.325–3.130)	1.000
Dizziness	289 (79.4)	75 (20.6)	3.853 (0.534–27.808)	0.194
Deep Vein Thrombosis	285 (78.9)	76 (21.1)	0.625 (0.074–5.270)	1.000
Osteoporosis	249 (77.8)	71 (22.2)	0.501 (0.205–1.226)	0.124
Osteoarthritis	225 (79.2)	59 (20.8)	1.040 (0.574–1.885)	0.897
Visual Impairment	184 (80.0)	46 (20.0)	1.159 (0.693–1.938)	0.574
Hearing Impairment	228 (79.4)	59 (20.6)	1.104 (0.608–2.006)	0.745
Urinary Incontinence	160 (81.2)	37 (18.8)	1.320 (0.798–2.184)	0.278
Fecal Incontinence	272 (79.5)	70 (20.5)	1.432 (0.579–3.541)	0.435
COPD (¥)	262 (79.4)	68 (20.6)	1.196 (0.541–2.645)	0.659
Previous Fracture	278 (79.4)	72 (20.6)	1.485 (0.513–4.301)	0.550

(*) Statistically Significant at P ≤ 0.05

(**) Highly Statistically Significant at P ≤ 0.01

(#) Fisher Exact Test was used when (25.0%) of the cells or more have Expected Count less than 5

(†) Haldane–Anscombe 0.5 was added for zero cells

(¥) Chronic Obstructive Pulmonary Disease

Association Between Medication Use and Falls shows that antipsychotics (OR = 2.61, 95% CI 1.24–5.49, *p* = 0.009) and antidepressants (OR = 2.79, 95% CI 1.58–4.92, *p* < 0.001) are associated with higher odds of falls, whereas most other medication classes (loop diuretics, thiazides, beta-blockers, calcium-channel blockers, oral hypoglycemics, insulin) show no statistically significant association. Antihypertensives (any) display a nonsignificant trend toward higher odds (OR = 1.58, 95% CI 0.94–2.68, *p* = 0.085), and anticoagulants trend toward lower odds (OR = 0.51, 95% CI 0.25–1.04, *p* = 0.062) (Table 5).

**Table 5:** Association between medication use and falls (*n* = 368)

Medication Class	No Falls *n* (%)	Falls *n* (%)	Odds Ratio (95% CI)	*P*-value (#)
Loop Diuretics	181 (78.0)	51 (22.0)	0.839 (0.495–1.423)	0.514
Thiazide Diuretics	255 (79.9)	64 (20.1)	1.439 (0.721–2.871)	0.300
Antihypertensive Medications	130 (83.3)	26 (16.7)	1.584 (0.936–2.680)	0.085
Anticoagulant Medications	225 (77.1)	67 (22.9)	0.509 (0.248–1.044)	0.062
Beta-Blockers	181 (77.7)	52 (22.3)	0.791 (0.464–1.348)	0.388
Alpha-Adrenergic Blockers	261 (79.6)	67 (20.4)	1.299 (0.605–2.789)	0.502
Calcium Channel Blockers	201 (80.1)	50 (19.9)	1.206 (0.710–2.049)	0.488
Anti-Parkinson Medications	279 (78.8)	75 (21.2)	0.620 (0.136–2.831)	0.743
Coagulation Enhancers	259 (79.4)	67 (20.6)	1.208 (0.565–2.581)	0.625
Antipsychotic Medications	270 (80.8)	64 (19.2)	2.612 (1.242–5.493)	0.009**
Antidepressant Medications	246 (82.8)	51 (17.2)	2.787 (1.577–4.924)	0.000**
Skeletal Muscle Relaxants	285 (79.4)	74 (20.6)	1.926 (0.470–7.882)	0.403
Antihistamines	261 (78.9)	70 (21.1)	0.870 (0.367–2.064)	0.752
Antiepileptic Medications	271 (79.0)	72 (21.0)	0.941 (0.341–2.594)	0.906
Anticholinergic Medications	277 (79.4)	72 (20.6)	1.374 (0.479–3.940)	0.564
Oral Hypoglycemic Agents	182 (77.8)	52 (22.2)	0.803 (0.471–1.368)	0.418
Insulin Therapy	197 (78.5)	54 (21.5)	0.893 (0.517–1.542)	0.684
Systemic Corticosteroids	285 (79.2)	75 (20.8)	1.267 (0.251–6.403)	0.675
Opioid Analgesics	290 (79.0)	77 (21.0)	1.249 (0.050–30.975) (†)	1.000

(**) Highly Statistically Significant at P ≤ 0.01

(#) Fisher Exact Test was used when (25.0%) of the cells or more have Expected Count less than 5

(†) Haldane–Anscombe 0.5 was added for zero cells

Comparison of Clinical and Laboratory Measures Between Fallers and Non-Fallers reveal that most measures were not significantly different between fallers and non-fallers (age, erect/supine BP, ADL, vitamin D, corrected calcium, TSH, hemoglobin, creatinine; all *p* ≥ 0.053), with two near-threshold trends—hemoglobin slightly lower in fallers (*p* = 0.053) and corrected calcium slightly lower (*p* = 0.075) (Table 6).

**Table 6:** Comparison of clinical and laboratory measures between fallers and non-fallers (*n* = 368)

Variable	No Falls Mean ± SD	Falls Mean ± SD	*P*-value (#)
Age (years)	82.32 ± 10.18	81.25 ± 8.38	0.394
Systolic Blood Pressure (Standing)	118.65 ± 14.64	120.47 ± 17.00	0.349
Diastolic Blood Pressure (Standing)	71.80 ± 10.73	70.91 ± 9.76	0.508
Systolic Blood Pressure (Supine)	120.90 ± 14.89	120.91 ± 14.97	0.995
Diastolic Blood Pressure (Supine)	72.92 ± 9.50	71.56 ± 9.88	0.268
Activities of Daily Living Score	3.10 ± 2.04	2.77 ± 1.93	0.198
Vitamin D Level	26.63 ± 15.59	27.94 ± 11.33	0.489
Corrected Calcium Level	2.20 ± 0.20	2.16 ± 0.16	0.075
Thyroid Stimulating Hormone	2.78 ± 2.85	2.25 ± 1.44	0.115
Hemoglobin Level	13.07 ± 1.95	12.55 ± 2.48	0.053
Serum Creatinine Level	62.72 ± 25.85	57.95 ± 24.01	0.145

(#) Independent sample t-test was used to compare between 2 independent group means

Relationship between 10-Fall Risk Assessment Tool score and occurrence of falls shows that higher fall-risk level was associated with a greater likelihood of falls: compared with the Low-risk group (17.5% falls), the Medium-risk group had a higher fall proportion (27.2%) with an odds ratio of 1.77 (95% CI: 1.04–2.99). The High-risk group (100% falls; zero non-falls observed) showed a markedly elevated odds ratio of 23.43 (95% CI: 1.11–496.46); however, this estimate should be interpreted cautiously due to the very small number of participants in this category, the presence of zero-event cells, and the resulting extremely wide confidence interval, indicating limited precision and potential instability of the estimate. The overall association by Fisher’s exact test was statistically significant (statistic = 10.020, *p* = 0.004). Moreover, the mean Fall Risk Score was significantly higher among those who fell compared with those who did not (5.00 ± 1.68 vs. 4.35 ± 1.53; independent-samples t = − 3.221, *p* = 0.001). Nevertheless, although these findings suggest a trend of increasing fall occurrence with higher risk scores, the magnitude of effect for the high-risk category should not be overemphasized given the sparse data (Table 7).

**Table 7:** Relationship between fall-risk level and fall occurrence (*n* = 368)

Categorical Fall-Risk Level				
Fall-Risk Level	No Falls *n* (%)	Falls *n* (%)	Odds Ratio (95% CI)	*P*-value (#)
Low Risk (Reference)	208 (82.5)	44 (17.5)	Reference	0.004**
Medium Risk	83 (72.8)	31 (27.2)	1.77 (1.04–2.99)	
High Risk	0 (0.0)	2 (100.0)	23.43 (1.11–496.46)	

**Continuous Fall-Risk Score**			
**Variable**	**No Falls** **Mean ± SD**	**Falls** **Mean ± SD**	***P*** **-value (##)**
Fall-Risk Score	4.35 ± 1.53	5.00 ± 1.68	0.001**

(#) Fisher’s exact test was used for comparison of fall-risk categories

(##) Independent-samples t-test was used for comparison of mean scores

**Highly statistically significant at *p* < 0.01

Concerning the relationship between Home Safety Assessment (STEADI) and falls, the present study shows a statistically significant overall association (Fisher’s exact = 15.993; *p* = 0.039), driven mainly by higher odds for Broken/Uneven/Loose steps (OR = 3.26; 95% CI: 1.09–9.76) and Missing/Insufficient handrails (OR = 20.58; 95% CI: 0.95–444.59). However, the estimate for missing/insufficient handrails should be interpreted with caution, as it is based on sparse data with zero-event cells and an extremely wide confidence interval, indicating substantial statistical imprecision and instability of the effect size. Other stair-related categories (e.g., “No hazard,” “Objects on stairs,” “No stairs,” “Loose carpet/throw rugs/slippery floor”) showed ORs near unity with non-significant differences compared to the reference category (“Not used”). Additionally, several categories contained very small cell counts (e.g., “Poor lighting/No light”), resulting in wide confidence intervals and reduced precision of the estimates.

In contrast, floors, bathrooms, bedrooms, and kitchen domains showed no significant overall association with falls (Fisher’s exact *p* = 0.341, 0.314, 0.483, and 0.114, respectively), and their category-specific ORs generally had wide confidence intervals crossing 1 (e.g., “Wires/cords present,” OR = 3.58; 95% CI: 0.49–26.11; “Objects on floor,” OR = 7.17; 95% CI: 0.64–80.73; “Dark path/Hard-to-reach lights,” OR = 12.10; 95% CI: 0.48–302.27; “Step stool sturdy,” OR = 7.80; 95% CI: 0.30–200.72). These wide intervals reflect limited event numbers in certain categories and indicate considerable statistical uncertainty. Some categories suggested potential lower odds (e.g., High shelves in kitchen, OR = 0.26; 95% CI: 0.03–2.23; Crowded/obstructive furniture on floors, OR = 0.56; 95% CI: 0.24–1.31), but none reached statistical significance.

Overall, while stair-related hazards—particularly defective steps and absent/insufficient handrails—appear to be the most notable environmental correlates in this dataset, these findings should be regarded as exploring and hypothesis-generating due to sparse data, small cell counts, and wide confidence intervals. Confirmation in larger, adequately powered studies is warranted before drawing definitive conclusions (Table 8).

**Table 8:** Home safety assessment (STEADI): relationship between specific household hazards and falls (*n* = 368)

Home Safety Assessment		Falls		OR (95% CI)	*P*-value (#)
		No Falls *n *(%)	Falls *n* (%)		
Stairs	Not used	88 (80.7)	21 (19.3)	Reference	0.039*
	No Hazard	150 (80.2)	37 (19.8)	1.03 (0.57–1.88)	
	No Stairs	13 (76.5)	4 (23.5)	1.29 (0.38–4.36)	
	Objects on Stairs	11 (78.6)	3 (21.4)	1.14 (0.29–4.46)	
	Broken/Uneven/Loose steps	9 (56.2)	7 (43.8)	3.26 (1.09–9.76)	
	Missing/Insufficient Handrails	0 (0.0)	2 (100.0)	20.58 (0.95-444.59)	
	Poor lightening/ No Light	12 (100.0)	0 (0.0)	0.16 (0.01–2.89)	
	Loose Carpet/ throw Rugs/ Slippery Floor	5 (71.4)	2 (28.6)	1.68 (0.30–9.24)	
	Unsafe (General)	2 (100.0)	0 (0.0)	0.82 (0.04–17.78)	
	Shoes on Stairs	1 (50.0)	1 (50.0)	4.19 (0.25–69.77)	
Floors	No Hazard	172 (78.2)	48 (21.8)	Reference	0.341
	Throw Rugs/ Loose Rugs	51 (79.7)	13 (20.3)	0.91 (0.46–1.82)	
	Wires/ Cords Present	2 (50.0)	2 (50.0)	3.58 (0.49–26.11)	
	Crowded/ Obstructive Furniture	45 (86.5)	7 (13.5)	0.56 (0.24–1.31)	
	Slippery Floor	9 (75.0)	3 (25.0)	1.19 (0.31–4.59)	
	Objects on Floor (Papers/Shoes/Things)	1 (33.3)	2 (66.7)	7.17 (0.64–80.73)	
	Support Needed	9 (81.8)	2 (18.2)	0.80 (0.17–3.81)	
	Torn/Damaged Carpet	1 (100.0)	0 (0.0)	1.19 (0.05–29.57)	
	Oxygen Tubing Present	1 (100.0)	0 (0.0)	1.19 (0.05–29.57)	
	Not used/ Not Applicable	0 (0.0)	0 (0.0)		
Bathrooms	No Hazard	132 (81.0)	31 (19.0)	Reference	0.314
	Slippery Floor Only	59 (80.8)	14 (19.2)	1.01 (0.50–2.04)	
	Needs Grab Bars/Support Only	41 (73.2)	15 (26.8)	1.56 (0.77–3.17)	
	Slippery + Needs Grab Bars/Support	59 (78.7)	16 (21.3)	1.15 (0.59–2.27)	
	Not Used/ Not Applicable	0 (0.0)	1 (100.0)	12.62 (0.50-317.15)	
Bedrooms	No Hazard	175 (80.3)	43 (19.7)	Reference	0.483
	No Bedside Light	65 (79.3)	17 (20.7)	1.06 (0.57-2.00)	
	Hard to Reach Lights	30 (73.2)	11 (26.8)	1.49 (0.69–3.21)	
	Dark path/ Hard-to-Reach Lights	0 (0.0)	1 (100.0)	12.10 (0.48-302.27)	
	Not Used/ Not Applicable	2 (100.0)	0 (0.0)	0.81 (0.04–17.11)	
	Lightening Issue (Unspecified)	15 (75.0)	5 (25.0)	1.36 (0.47–3.94)	
	Other Hazard (Un Specified)	4 (100.0)	0 (0.0)	0.45 (0.02–8.48)	
Kitchen	No Hazard	45 (72.6)	17 (27.4)	Reference	0.114
	Not Used/ NA	236 (80.3)	58 (19.7)	0.65 (0.35–1.22)	
	High Shelves (Storage out of Reach)	10 (90.9)	1 (9.1)	0.26 (0.03–2.23)	
	Step Stool Sturdy (Mitigation Present)	0 (0.0)	1 (100.0)	7.80 (0.30-200.72)	

(*) Statistically Significant at *P*≤ 0.05

(#) Fisher Exact Test was used when (25.0%) of the cells or more have Expected Count less than 5

The multivariable logistic regression analysis demonstrated that, after adjusting for potential confounders, antidepressant use, and higher fall risk scores remained independently associated with increased odds of fall occurrence among older adults receiving home healthcare services. Specifically, participants using antidepressants had more than twice the odds of experiencing a fall (AOR = 2.52, 95% CI: 1.20–5.26, *p* = 0.014), while each unit increase in fall risk score was associated with a 22% increase in fall odds (AOR = 1.22, 95% CI: 1.03–1.45, *p* = 0.018). Increasing age showed a modest but statistically significant inverse association with falls (AOR = 0.98, 95% CI: 0.97–1.00, *p* = 0.008), and female sex was associated with lower odds of falling compared to males (AOR = 0.43, 95% CI: 0.27–0.71, *p* = 0.001). In contrast, stroke, depression, antipsychotic use, and stair-related environmental hazards were not independently associated with falls after adjustment. It should be noted that some variables demonstrated relatively wide confidence intervals, reflecting limited event numbers in certain categories; therefore, the magnitude of both significant and non-significant estimates should be interpreted cautiously and not overemphasized. The model demonstrated good fit (Hosmer–Lemeshow *p* = 0.832) and explained approximately 48.8% of the variance in fall occurrence (Nagelkerke R² = 0.488), indicating acceptable explanatory power (Table 9).

**Table 9:** Multivariable logistic regression analysis of factors independently associated with fall occurrence among older adults receiving home healthcare services (*n* = 368)

Variable	Adjusted OR (95% CI)	*p*-value
Age	0.98 (0.97–1.00)	0.008**
Female sex	0.43 (0.27–0.71)	0.001**
Stroke	1.58 (0.89–2.79)	0.116
Depression	0.98 (0.43–2.24)	0.97
Antipsychotics	1.58 (0.70–3.59)	0.271
Antidepressants	2.52 (1.20–5.26)	0.014*
Fall Risk Score	1.22 (1.03–1.45)	0.018*
Stairs (Environmental Hazard)	1.10 (0.95–1.27)	0.202

(*) Statistically Significant at *P* **≤**0.05

(**) Highly Statistically Significant at *P* **≤** 0.01

Model Fit Statistics: −2 Log Likelihood: 342.56, Cox & Snell R²: 0.366, Nagelkerke R²: 0.488, Hosmer–Lemeshow Test: χ² = 4.26, *p* = 0.832

## Discussion

Falls are a major geriatric concern, particularly among the old–old and oldest-old and are commonly observed in the context of age-related declines in balance, strength, and physiological reserve, as well as the higher prevalence of multimorbidity and functional limitations. The present study revealed that our predominantly very old sample (mean age 82.1 years, 71.2% women) had a 12-month fall prevalence of 20.9%, a figure that aligns with recent global estimates reporting annual fall rates of 20–30% among community-dwelling older adults [2, 16]. This relatively modest prevalence, despite the advanced age structure, parallels findings from China and Europe showing similarly wide ranges (8.6–39.7%) in very old populations [17, 18].

However, interpretation of these findings should consider the study setting, as participants were recruited from a tertiary, military-affiliated hospital-based home healthcare program, which primarily serves retired military personnel and their eligible dependents. This population may differ from the general older population in terms of healthcare access, structured follow-up, and continuity of care, potentially influencing fall detection, reporting patterns, and preventive practices. The comparable prevalence observed despite higher clinical complexity suggests that structured, physician-led home healthcare services may play a stabilizing role in mitigating fall occurrence, even among highly vulnerable individuals. This finding contributes new insight by indicating that fall prevalence in medically complex home healthcare populations may not necessarily exceed community estimates when systematic monitoring and multidisciplinary oversight are in place.

Among fallers, most experienced a single fall (76.6%), while 23.4% had recurrent falls, a pattern consistent with Malaysian longitudinal data in which roughly one quarter to one third of fallers progress to recurrent events, often observed alongside emerging frailty [16]. Furthermore, the low population-level injury (5.2%) and fracture prevalence (2.7%) observed here are comparable with international reports indicating that 5–10% of falls are associated with serious injury and that approximately 3% of community-dwelling older adults sustain significant fall-related injuries annually [16, 17, 19]. It is possible that structured medical oversight within the Armed Forces Hospital home healthcare program contributed to earlier identification of risk factors or timely management of comorbidities, which may influence injury severity patterns. Importantly, the combination of advanced age, high multimorbidity burden, and relatively modest injury rates suggests that fall severity in this cohort may be influenced not only by intrinsic vulnerability but also by contextual healthcare factors, including rapid response, caregiver supervision, and ongoing clinical surveillance. This contextual dimension adds to the literature by highlighting the interaction between healthcare delivery structure and fall outcomes.

Overall, these findings suggest that although fall and injury rates in this cohort fall within global expectations, recurrent fallers—typically very old and predominantly female—represent a subgroup in whom fall occurrence is more frequently observed, warranting targeted prevention efforts [16, 18, 20]. Nevertheless, caution is warranted when generalizing these results to other healthcare settings in Saudi Arabia, particularly Ministry of Health or private-sector institutions that may serve populations with different sociodemographic and clinical characteristics. The present study therefore contributes setting-specific evidence from a hospital-based home healthcare model in Saudi Arabia, addressing a national evidence gap and providing data that may inform context-adapted fall prevention strategies within similar structured home healthcare systems.

The present study highlights a pronounced multimorbidity burden dominated by cardiometabolic disease, with very high rates of hypertension (80.4%) and diabetes (74.5%), consistent with recent international data showing these conditions as the most common disorders in adults ≥ 75 years [21, 22]. Cardiovascular morbidity—including ischemic heart disease, atrial fibrillation, and prior stroke—was also substantial and aligns with global geriatric studies reporting vascular disease in up to half of the oldest-old [23]. Non-cardiac conditions such as chronic kidney disease, osteoarthritis, osteoporosis, hypothyroidism, depression, and dementia were similarly frequent, reflecting patterns observed in large aging cohorts where multimorbidity exceeds 70% [21, 24]. Sensory and functional impairments, especially visual loss, hearing impairment, and urinary incontinence, were also common and have been reported to be associated with mobility decline and fall occurrence in population-based studies [25]. Less common conditions (e.g., hyperthyroidism, deep vein thrombosis, anemia, Parkinson’s disease) followed expected epidemiological trends, indicating that the overall health profile of this cohort aligns with global patterns of aging characterized by heavy cardiometabolic, vascular, musculoskeletal, endocrine, and sensory burdens [20–25].

Importantly, the exceptionally high prevalence of cardiometabolic disorders in this home healthcare cohort reflects not only age-related disease accumulation but also the clinical eligibility profile of patients enrolled in hospital-based home healthcare services, who typically require ongoing medical monitoring and chronic disease management. This concentration of multimorbidity may contribute to cumulative vulnerability through mechanisms such as orthostatic instability, polypharmacy, sarcopenia, and reduced physiological reserve. Thus, the study adds context-specific evidence by illustrating how multimorbidity clusters within structured home healthcare programs may shape fall-related risk patterns differently from relatively independent community-dwelling populations.

The present study indicates that although a very high proportion of participants were unable to stand after a fall (90.2%), the rate of serious post-fall complications was relatively low, with fractures occurring in only 2.4%, hematomas in 1.4%, and hospitalizations in 0.8%, and no cases of subdural hematoma reported. These findings are consistent with recent community-based research showing that while difficulty standing or requiring assistance after a fall is common among frail older adults, major injuries are reported in a smaller fraction—typically 5–10% for serious injury and 1–3% for fractures [26, 27]. Studies from China and Canada similarly report low rates of fall-related hospitalization and intracranial injury in community settings, despite high functional vulnerability among fallers [17, 27].

The marked discrepancy between the high inability-to-rise rate and the relatively low injury burden is particularly noteworthy. It suggests that in this cohort, post-fall immobility may reflect functional frailty, impaired lower-extremity strength, or balance deficits rather than severe traumatic injury. Within a structured home healthcare context, timely caregiver assistance and rapid clinical evaluation may further limit progression to severe complications. This observation provides an important clinical insight. It highlights that inability to stand after a fall in home healthcare recipients may serve as a functional vulnerability marker rather than a direct indicator of injury severity, reinforcing the need for functional assessment and rehabilitation-focused interventions.

Overall, the low prevalence of serious injuries in the present study aligns with international evidence, but the functional consequence of being unable to rise after a fall underscores the importance of prompt assessment, assistance, and preventive strategies even when clinically significant trauma is uncommon [27, 28]. Collectively, these findings emphasize that fall-related risk in home healthcare populations may be driven more by functional frailty and chronic disease interaction than by high-impact trauma alone, thereby informing tailored fall prevention strategies that integrate medical optimization with strength and mobility support.

The present study found that prior stroke and depression were significantly associated with increased fall occurrence in univariate analyses, with stroke nearly doubling the odds of falling and depression showing a comparable magnitude of association. These findings align with recent literature in which stroke-related motor deficits, impaired balance, and residual neurological dysfunction have been reported to be associated with higher fall occurrence among older adults, with several cohort studies documenting 1.5–3-fold higher odds of falls post-stroke [29]. Likewise, depression has consistently been associated with fall occurrence, potentially through mechanisms such as impaired concentration, psychomotor slowing, reduced physical activity, and the use of antidepressant medications; meta-analyses report that depressive symptoms are associated with approximately 50–70% higher odds of falls [30].

However, in the multivariable logistic regression model, neither stroke nor depression remained independently associated with falls after adjustment for age, sex, medication use, and fall-risk score. Therefore, these associations should be interpreted cautiously, as they may partly reflect confounding by coexisting clinical factors rather than independent effects. This attenuation after adjustment suggests that in this home healthcare population, the apparent effect of stroke and depression may operate indirectly through functional vulnerability, medication exposure, or cumulative risk captured by the composite fall-risk score, rather than representing isolated disease-specific effects. This finding adds nuance to existing literature by highlighting that, within medically supervised home healthcare settings, the interplay between comorbidity, medication use, and overall frailty may be more informative than single-disease labels when evaluating fall patterns.

In contrast, most other comorbidities in our study—including cardiometabolic disorders, musculoskeletal problems, sensory impairments, incontinence, thyroid disease, chronic obstructive pulmonary disease, anemia, dizziness, seizures, and prior fractures—were not statistically associated with falls, a pattern that agrees with large epidemiologic studies showing that multimorbidity alone is often less strongly associated with falls than specific high-risk conditions affecting neuromuscular, cognitive, or affective function [31, 32]. Importantly, this observation suggests that within structured home healthcare programs—where chronic conditions are routinely monitored and medically managed—the mere presence of multiple diseases may not sufficiently discriminate fall risk without accompanying functional or neurocognitive impairment. This selective association may indicate that, within this cohort, fall occurrence was more frequently observed among individuals with conditions affecting neurological control, mood regulation, and functional mobility rather than reflecting the overall burden of chronic diseases [30–32].

The present study shows that antipsychotics and antidepressants were significantly associated with increased fall occurrence in univariate analyses, with both more than doubling the odds of falling. Psychotropic medications have been reported to be associated with impaired alertness, slower reaction time, gait instability, and postural control, and numerous meta-analyses identify among the medication classes most consistently associated with falls in older adults [33, 34]. However, after multivariable adjustment, only antidepressant use remained independently associated with fall occurrence, whereas the association with antipsychotics was attenuated and no longer statistically significant.

The persistence of the antidepressant association after adjustment may reflect a combined influence of underlying depressive symptoms, residual psychomotor effects, and potential orthostatic or sedative properties of certain agents. Conversely, the attenuation of the antipsychotic association suggests that its apparent univariate effect may be partly explained by coexisting cognitive impairment, behavioral symptoms, or greater baseline frailty among users. These findings underscore the importance of distinguishing between medication effects per se and the clinical context in which these medications are prescribed.

Collectively, this study contributes setting-specific evidence indicating that in a hospital-affiliated home healthcare population, antidepressant use may represent a more robust marker of fall vulnerability than other psychotropic classes after accounting for demographic and clinical covariates. Nonetheless, given the cross-sectional design, these findings should be interpreted as associative rather than causal, and prospective studies are needed to clarify temporal relationships between medication exposure and fall events.

In contrast, most other commonly used drugs—such as loop and thiazide diuretics, beta-blockers, calcium-channel blockers, oral hypoglycemic agents, and insulin—did not show statistically significant associations in this sample, aligning with recent studies suggesting that these medications are not consistently associated with increased fall occurrence when appropriately prescribed and monitored [35]. This finding may reflect the structured follow-up and medication reconciliation processes within the hospital-affiliated home healthcare program, where dosing adjustments and monitoring for adverse effects are routinely performed. Antihypertensive medications showed a non-significant trend toward higher odds of falling, consistent with mixed evidence indicating that while antihypertensive therapy may be associated with orthostatic symptoms in some individuals, contemporary trials generally do not demonstrate a robust association with falls [36]. Interestingly, anticoagulants trended toward lower fall occurrence, a finding also reported in recent analyses suggesting that anticoagulation is not consistently associated with increased falls and may reflect differences in patient characteristics, closer clinical supervision, or selective prescribing practices [37].

Overall, the findings suggest that only antidepressant use demonstrated a consistent independent association with falls in this cohort, whereas most cardiometabolic medications appear to show limited or context-dependent associations [33–37]. This distinction is clinically relevant, as it suggests that in medically supervised home healthcare populations, medication-related fall vulnerability may be more strongly linked to neurocognitive or psychotropic effects than to cardiometabolic pharmacotherapy alone.

The present study also shows that most clinical and laboratory parameters—including age, blood pressure (supine and erect), activities of daily living function, vitamin D levels, corrected calcium, thyroid-stimulating hormone, hemoglobin, and creatinine—did not differ significantly between fallers and non-fallers, indicating that routine physiological and biochemical measures were not significantly associated with fall occurrence in this sample. These findings align with recent geriatric evidence showing that standard laboratory values often fail to distinguish fallers from non-fallers, as functional, neurological, and medication-related factors tend to demonstrate stronger associations with fall occurrence [1, 38].

Notably, the absence of strong laboratory associations in this relatively medically supervised cohort may suggest that biochemical abnormalities are either adequately managed or insufficient alone to precipitate falls without concurrent functional impairment. The near-significant trends observed—slightly lower hemoglobin (*p* = 0.053) and slightly lower corrected calcium (*p* = 0.075) in fallers—may reflect potential associations that did not reach statistical significance in this sample and are consistent with prior studies in which anemia and subtle disturbances in calcium homeostasis have been reported to be associated with impaired muscle strength, balance instability, and falls [39, 40]. However, given the cross-sectional design and modest effect sizes, these trends should be interpreted cautiously and warrant confirmation in larger prospective studies.

Overall, the results support the observation that laboratory abnormalities may show modest associations with fall vulnerability but appear less consistently associated than clinical factors such as neurological disease, depression, psychotropic medication use, and cumulative fall-risk burden [1–40]. This reinforces the importance of prioritizing multidimensional functional assessment over isolated laboratory markers when evaluating fall patterns in home healthcare populations.

The present study shows a strong, graded association between the Missouri Alliance for Home Care’s 10-Fall Risk Assessment Tool score and actual fall occurrence, with fall proportions rising from 17.5% in the low-risk group to 27.2% in the medium-risk group (OR = 1.77) and reaching 100% in the high-risk group (OR = 23.43). However, the estimate for the high-risk category was based on a very small number of participants and zero-event cells, resulting in a wide confidence interval; therefore, the magnitude of this association should be interpreted cautiously.

Importantly, the observed gradient across risk categories suggests that the tool captures clinically meaningful clustering of vulnerability factors within this home healthcare population, even in a cross-sectional framework. Unlike many validation studies conducted in community-dwelling cohorts, our findings demonstrate that structured fall-risk stratification remains informative in a medically complex, functionally dependent home healthcare setting. This pattern aligns with recent evidence demonstrating that multifactorial fall-risk stratification has been reported to be associated with higher subsequent fall occurrence in longitudinal studies, as highlighted in the 2022 World Guidelines for Falls Prevention [37]. Recent studies using composite or artificial intelligence–derived fall-risk scores also report clear dose–response relationships, where higher scores are associated with higher fall rates [41, 42].

The contribution of the present study lies in extending this evidence to a Saudi Arabian hospital-based home healthcare context, where structured geriatric assessment tools are not uniformly standardized across institutions. Furthermore, contemporary reviews confirm that although predictive accuracy varies among tools, higher numerical risk categories generally identify individuals with greater observed fall occurrence [43, 44]. Overall, the findings support the clinical utility of structured fall-risk scoring, showing that higher risk levels are associated with increased likelihood of falling in this sample [41–44]. Nevertheless, given the cross-sectional nature of the study, these findings reflect association rather than predictive causality.

The present study indicates a statistically significant overall association between the STEADI Home Safety Assessment and fall occurrence (Fisher’s exact = 15.993; *p* = 0.039), driven predominantly by stair-related hazards—specifically broken, uneven, or loose steps (OR = 3.26) and missing or insufficient handrails (OR = 20.58). However, these effect estimates—particularly for missing or insufficient handrails—were accompanied by wide confidence intervals and sparse data in certain categories, indicating limited precision and statistical instability.

The prominence of stair-related hazards in this cohort may reflect the interaction between environmental structure and functional frailty. Older adults receiving home healthcare services often have reduced lower-limb strength, impaired balance, and slower reaction times; therefore, even minor structural stair defects may disproportionately influence fall occurrence in this subgroup. This pattern is generally consistent with recent evidence in which defects in stair structure and inadequate handrail support have been associated with higher fall occurrence, as stairs require higher balance, strength, and visual coordination than other household areas, and hazards on stairs may exacerbate mobility challenges in older adults [45]. However, because some stair-related categories contained small cell counts, these findings should be interpreted cautiously and considered exploratory rather than definitive.

In contrast, other environmental domains—floors, bathrooms, bedrooms, and kitchens—did not show significant associations, consistent with studies reporting that general home clutter, poor lighting, cords, and loose rugs often demonstrate weaker or inconsistent associations with falls after adjusting for personal risk factors such as mobility and strength [46]. One possible explanation is that in structured home healthcare programs, routine caregiver involvement and environmental awareness may mitigate some lower-level hazards, thereby attenuating measurable associations. Although some categories in the present dataset suggested possible trends (e.g., objects on floors, dark pathways, high kitchen shelves), their wide confidence intervals and non-significant findings reflect variability, small cell counts and limited statistical precision. Recent analyses have similarly suggested that structural hazards (e.g., steps, handrails) may show more consistent associations than general room-level features [47].

Collectively, while stair-related hazards appear to be the most notable environmental correlates in this dataset, these findings should be interpreted as exploratory and hypothesis-generating rather than definitive. A key insight from this study is that within a medically supervised home healthcare population, structural environmental vulnerabilities may interact with functional impairment in ways that are not fully captured by traditional community-based fall models. Given the sparse counts in several categories and resulting wide confidence intervals, confirmation in larger, adequately powered studies is necessary before firm conclusions or policy recommendations can be made.

### Study limitations & strengths

This study has several important limitations that warrant careful consideration. First, the cross-sectional design inherently limits the ability to establish temporal relationships or causal inferences between associated factors and fall occurrence. Because exposure and outcome were assessed simultaneously, it is not possible to determine directionality, and reverse causation cannot be excluded. Accordingly, all identified relationships should be interpreted strictly as associations rather than determinants of falls.

Second, although multivariable logistic regression was performed, residual confounding cannot be excluded. Some associations observed in univariate analyses (e.g., stroke, depression, and antipsychotic use) did not remain statistically significant after adjustment, indicating potential confounding by age, sex, medication burden, or overall functional status. Furthermore, the limited number of fall events relative to some exposure categories may have reduced statistical power for detecting independent associations.

Third, the reliance on retrospective self-reported fall history over the previous 12 months introduces the potential for recall bias. This concern is particularly relevant in a very old population, where cognitive impairment is common and may affect recall accuracy, even though caregiver input and medical record verification were used when available. Additionally, minor, unwitnessed, or unreported falls may have resulted in underestimation of the true fall prevalence.

Moreover, the home safety assessment was conducted during a single visit and may not fully capture temporary, seasonal, or evolving environmental hazards. Several environmental categories—particularly stair-related hazards—involved sparse data and small cell counts, resulting in wide confidence intervals and reduced statistical precision of some odds ratio estimates. These imprecise estimates limit the robustness of effect size interpretation. Accordingly, the magnitude of associations—especially those accompanied by very wide confidence intervals—should be interpreted cautiously. Environmental findings, particularly those related to stair safety, should therefore be considered exploratory and hypothesis-generating rather than definitive, pending confirmation in larger and more adequately powered longitudinal studies.

Despite these limitations, the study has notable strengths. These include a relatively large and well-defined sample of older adults actively receiving home healthcare services, the use of validated assessment tools such as the Missouri Alliance for Home Care’s 10-Fall Risk Assessment Tool and the CDC STEADI assessment, a comprehensive evaluation of medical, medication-related, and environmental factors associated with falls, and real-world home-based data collection. These strengths enhance the internal consistency of the findings and support their clinical relevance within the context of home healthcare services.

## Conclusion and recommendations

Falls represent an important health concern among older adults receiving home healthcare services. In this cohort, fall occurrence was statistically associated with selected comorbidities, psychotropic medication use, higher fall-risk scores, and certain environmental characteristics—particularly stair-related hazards. However, given the cross-sectional design and sparse data in some environmental categories, these findings should be interpreted cautiously, and stair-related observations should be considered exploratory and hypothesis-generating. These observed associations suggest several practice-relevant considerations for home healthcare services. Routine use of structured fall-risk assessment tools may assist in identifying individuals with higher observed fall occurrence. Periodic medication review—especially of psychotropic medications such as antidepressants—may be considered within comprehensive geriatric care, guided by individualized clinical judgment. Incorporating structured home safety evaluations, with attention to stair integrity and handrail availability, may also be reasonable within routine assessments. Strengthening caregiver education and enhancing staff awareness of fall-associated clinical and environmental patterns may further support consistent implementation of fall-safety practices.

Importantly, these recommendations are informed by observed associations rather than demonstrated causal effects. Their effectiveness in reducing fall occurrence should be confirmed through prospective, longitudinal, and interventional research.

## Supplementary Information

Supplementary Material 1.

## Data Availability

The datasets generated and analyzed during the current study are not publicly available due to patient privacy and institutional policy restrictions, but are available from the corresponding author on reasonable request.

## Abbreviations

ADL: Activities of Daily Living
AF: Atrial Fibrillation
AFHSR: Armed Forces Hospital Southern Region
BMI: Body Mass Index
BP: Blood Pressure
Ca c: Corrected Calcium
CCBs: Calcium Channel Blockers
CDC: Centers for Disease Control and Prevention
CKD: Chronic Kidney Disease
COPD: Chronic Obstructive Pulmonary Disease
DM: Diabetes Mellitus
DVT: Deep Vein Thrombosis
FE: Fisher’s Exact (test)
HHC: Home Health Care
HTN: Hypertension
IBM: International Business Machines (SPSS software provider)
IL: Interstitial Lung Disease
IHD / ISHD: Ischemic Heart Disease
OR: Odds Ratio
SPSS: Statistical Package for the Social Sciences
STEADI: Stopping Elderly Accidents, Deaths, and Injuries
TSH: Thyroid–Stimulating Hormone
Vit D: Vitamin D
